# Spina Ventosa of the Right Index Finger in an Adult Indian Female With No Pulmonary Involvement: A Rare Case

**DOI:** 10.7759/cureus.42904

**Published:** 2023-08-03

**Authors:** Sankalp Yadav, Gautam Rawal, Madhan Jeyaraman

**Affiliations:** 1 Medicine, Shri Madan Lal Khurana Chest Clinic, Moti Nagar, New Delhi, IND; 2 Respiratory Medical Critical Care, Max Super Speciality Hospital, New Delhi, IND; 3 Orthopaedics, ACS Medical College and Hospital, Dr MGR Educational and Research Institute, Chennai, IND

**Keywords:** tuberculosis, cbnaat/ xpert/ rif assay, mtb (mycobacterium tuberculosis), spina ventosa, dactylitis

## Abstract

Tubercular involvement of small bones is a rare clinical condition. Often, the disease is associated with pulmonary involvement. Isolated cases of spina ventosa in adults with no pulmonary seeding or any history of tuberculosis or trauma are relatively infrequent occurrences. We report a case of a 22-year-old immunocompetent Indian female who presented with complaints of a swollen right index finger. A detailed clinical workup supported by radiographs and serological tests established the diagnosis as primary tubercular dactylitis of the right index finger. She was managed with anti-tubercular drugs.

## Introduction

Tuberculosis is the result of infection due to the inhalation of *Mycobacterium tuberculosis *[1]. It is a significant threat to public health systems and is a sizeable contributor to morbidity and mortality [2]. It is commonly present in developing countries [2].

Spina ventosa, or tubercular dactylitis, was first identified by Boyer in 1803, while the tuberculous etiology of this condition was proved by Nelaton in 1837 [3]. Rankin in 1886 diagnosed it by histological technique, and Feilchenfeld described it roentgenographically in children in 1896 [4]. It is a rare clinical presentation of extrapulmonary tuberculosis [3]. Further, spina ventosa is a broad term that means any lesion of the bone that results in intensifying cortical absorption around the medullary canal with a growing subperiosteal hyperplasia until radiological identification of inflated and destructed bone [3]. It is usually seen in <2-4% of all cases of skeletal tuberculosis [5].

A case of a young Indian female in her early 20s is presented. She came with a swollen right index finger and no other constitutional signs of tuberculosis. A detailed diagnostic workup established the diagnosis as primary tubercular dactylitis of the right index finger. She was initiated on anti-tubercular drugs.

## Case presentation

A 22-year-old Hindu, unmarried, non-diabetic female belonging to a low socioeconomic group came as a referral case from a private set-up with complaints of painful swelling involving her right index finger for four months. She informed us that initially this swelling was small but progressed to its present size during the last four months. There was no history of weight loss, cough, fever, or any other constitutional symptoms of tuberculosis. She was a domestic helper and had never smoked. Also, there was no history of trauma or tuberculosis in her or among her contacts. And there was no history of stays at refugee camps or night shelters.

A general examination revealed a hemodynamically stable female. She was medium-built, and there was no icterus, clubbing, cyanosis, pretibial edema, or pallor. Her systemic examination was unremarkable.

**Figure 1:**
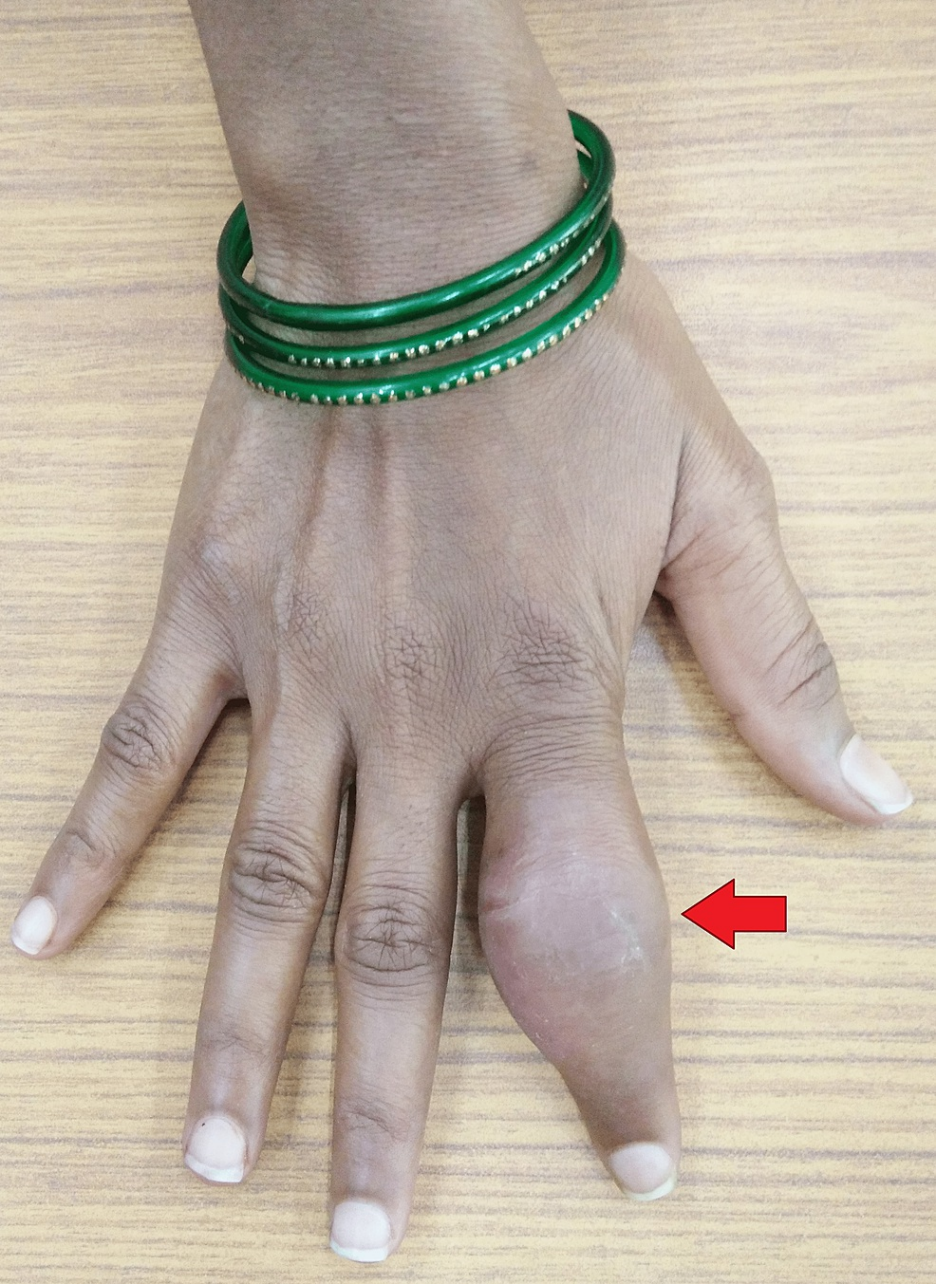
Gross image of right index finger showing fusiform swelling over the proximal and middle phalanges

**Figure 2:**
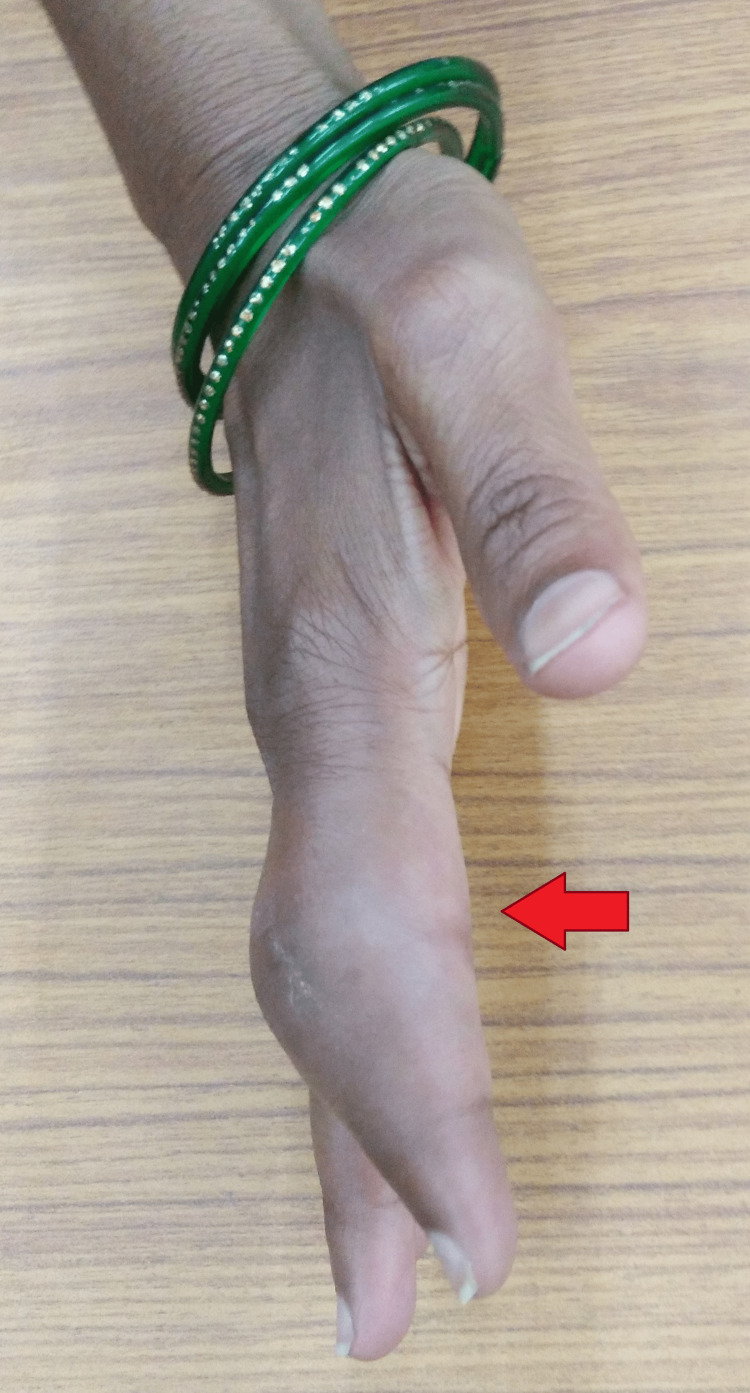
Gross image (lateral view) of right index finger showing swelling over the proximal and middle phalanges

Local examination of the right index finger revealed a visibly swollen, firm, and fusiform swelling over the proximal and middle phalanges. It was associated with overlying erythema. Movement was restricted at the distal and proximal interphalangeal joints. However, there were no discharging sinuses (Figures 1, 2).

**Figure 3:**
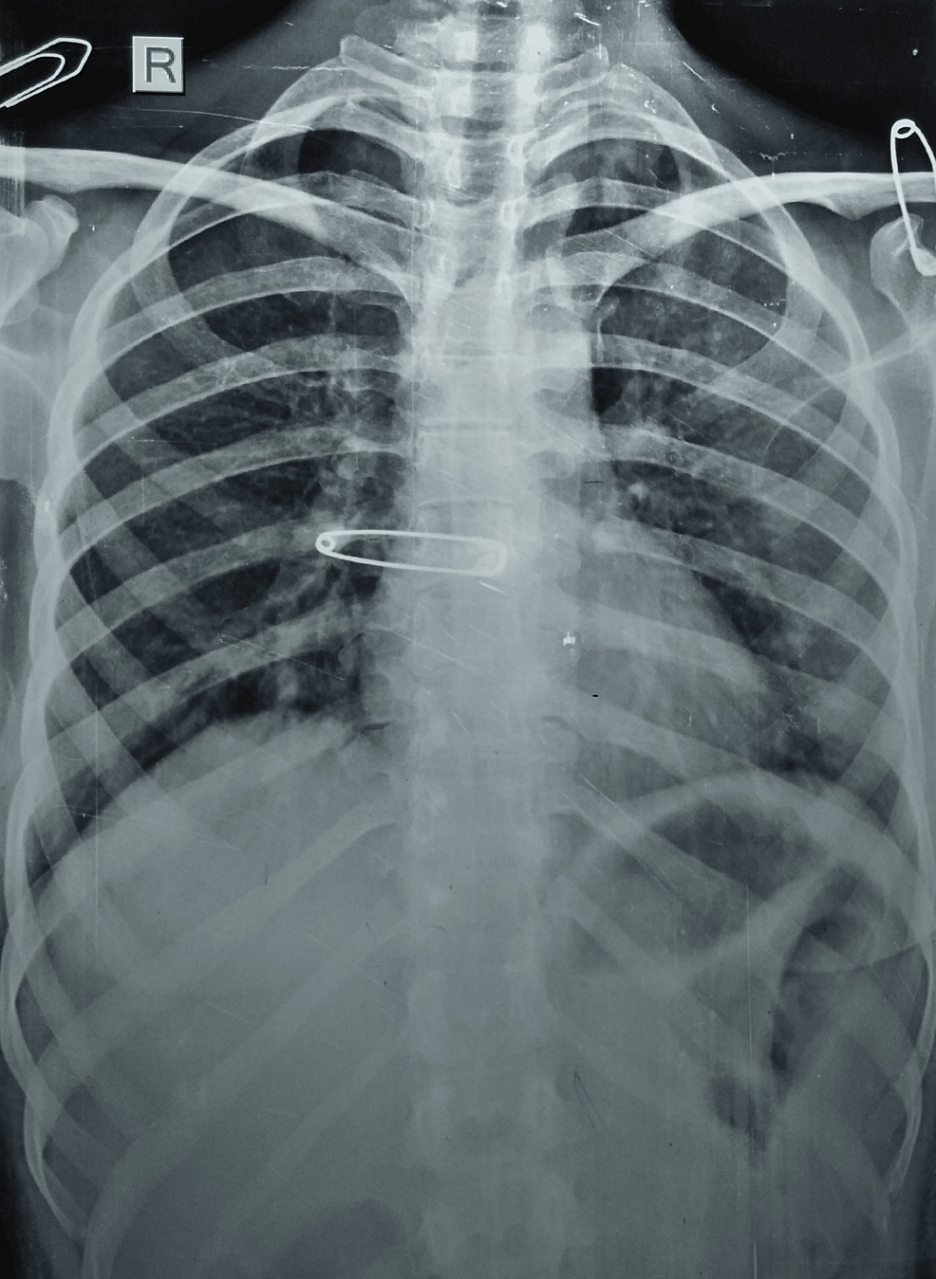
A chest radiograph (P-A view) not suggestive of tuberculosis P-A: postero-anterior

A preliminary diagnosis of chronic pyogenic osteomyelitis was made with a differential diagnosis of tuberculous dactylitis, syphilitic dactylitis, fungal dactylitis, and enchondroma. She was advised to undergo a series of serological tests and a radiograph of the chest and right hand. A chest radiograph was unremarkable for pulmonary disease (Figure 3).

Laboratory tests showed a raised erythrocyte sedimentation rate of 41 mm in the first hour with a hemoglobin of 11.0 g/dL. HIV, hepatitis panel (A, B, and C), and venereal disease research laboratory tests were negative. Her rheumatoid factor was negative, but her Mantoux test was strongly positive (31 mm).

**Figure 4:**
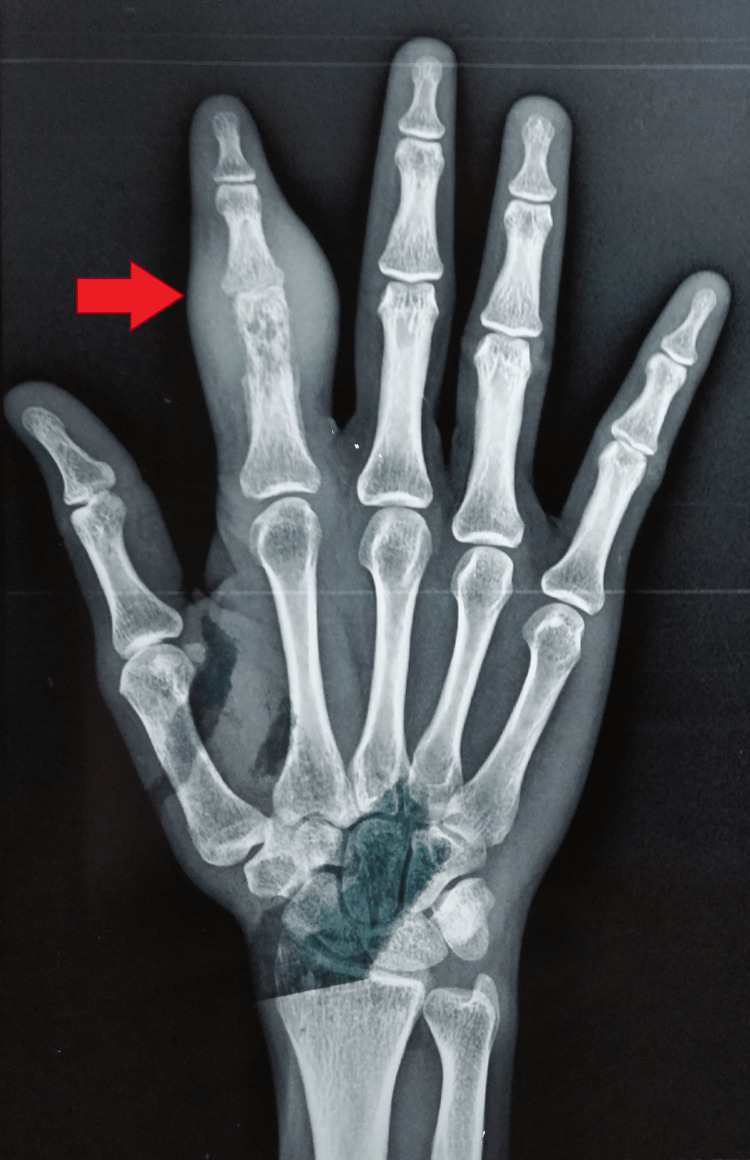
Radiograph of the right hand (P-A view) showing soft tissue swelling with cortical erosion in the distal part of the proximal phalanx P-A: postero-anterior

**Figure 5:**
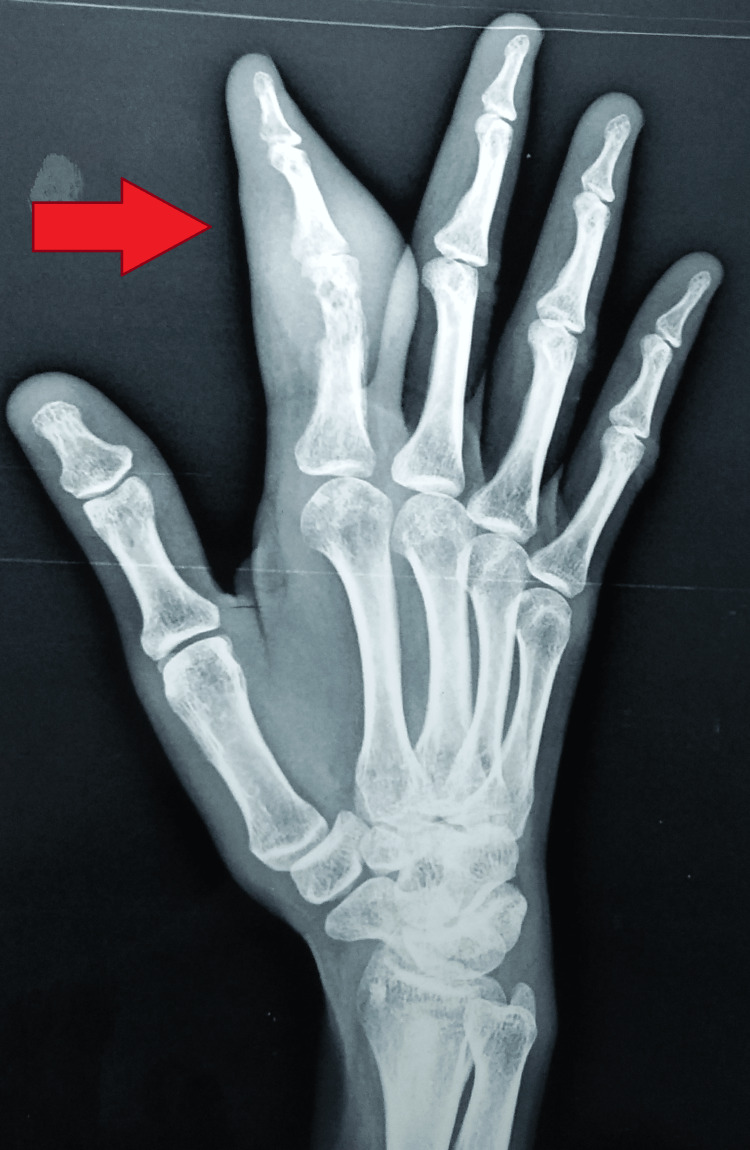
Radiograph of right hand oblique view showing cortical erosion in the distal part of proximal phalanx, and minimal periosteal reaction

The radiographs of the right hand showed soft tissue swelling with cortical erosion in the distal part of the proximal phalanx and minimal periosteal reaction (Figures 4, 5).

An ultrasound-guided biopsy was done, which showed granulomatous inflammation involving the dermis and subcutaneous fat, with necrosis and Langhans giant cells. Samples were also sent for Gram staining and culture for bacteria, fungi, and mycobacteria, but the results were negative. Another sample was sent for cartridge-based nucleic acid amplification testing and a line-probe assay. The results were positive, with detection of *Mycobacterium tuberculosis *on both, with no resistance to rifampicin or isoniazid. So, a final diagnosis of tubercular dactylitis of the distal part of the proximal phalanx of the right index finger was made, and she was put on anti-tubercular chemotherapy for 12 months (Table 1).

**Table 1:** Anti-tubercular chemotherapy for 12 months

Phase	Drug	Dose	Duration	Route
Intensive phase	Rifampicin	10 mg/kg	56 days	Per oral
	Pyrazinamide	25 mg/kg	56 days	Per oral
	Ethambutol	15 mg/kg	56 days	Per oral
	Isoniazid	5 mg/kg	56 days	Per oral
Continuation phase	Rifampicin	10 mg/kg	10 months	Per oral
	Ethambutol	15 mg/kg	10 months	Per oral
	Isoniazid	5 mg/kg	10 months	Per oral

A tablet of pyridoxine (1 mg/kg/day) was taken for the full duration of treatment, and counseling was done regarding dietary advice for a high-protein diet. After completion of the initiation phase, she was transferred to a different city at her request as she had lost her job due to the ongoing pandemic. However, she was advised for regular follow-up in the infectious diseases and orthopedics outpatient department of her native city but was lost to follow-up.

## Discussion

Tuberculosis manifesting at extrapulmonary sites is relatively rare (10-15% of all tuberculosis cases) [6]. Of all the extrapulmonary tuberculosis cases, skeletal involvement constitutes only 1-3% [5]. Of these, the spine and hip are the major contributors [5]. Tuberculosis of the small bones of the hand and feet is an infrequently reported disease [5]. It is commonly called tubercular dactylitis and is seen in 14% and 10% of all cases of skeletal tuberculosis [5,7]. Usually seen in children (about 85%) under the age of six years, this disease is rare in adults [8]. With a proclivity toward the hands more than the feet, this disease is reported to involve multiple sites in children, but in adults it is mostly localized to a single site [8]. The commonest bones involved are the proximal phalanx of the index and middle fingers [9]. There is a paucity of data related to this disease, mainly due to its indolent course and sparingly manifesting systemic clinical features [8].

In the absence of risk factors, spina ventosa is rare, especially after the age of five [5]. The disease mainly spreads by lympho-hematogenous spread to the small bones [8]. But direct infection post-trauma could also be linked to this condition [10]. Diagnosis is often delayed as there is an absence of constitutional symptoms (with no evidence of active pulmonary disease in >50% of patients) of tuberculosis in the majority of cases, which results in a late presentation [5]. The paucibacillary nature of the disease, a lack of awareness among the treating clinicians, overlapping clinical features with other musculoskeletal diseases, and nonspecific appearances on multimodal imaging are other factors [8]. Often the initial presentation is a soft tissue swelling and periostitis, which subsequently develop into expansile bony destruction and sequestrum formation [8].

Management is essentially medical, with the use of anti-tubercular drugs [5]. The national guidelines recommend treatment for 12 months, as advised in this case [11]. Any further extension of treatment depends upon the condition of the patient at the end of one year [11].

There is a paucity of data about the spina ventosa of the index finger in adults [12]. A similar case was presented by Bishnoi and Kumaran, where a 21-year-old female presented with similar clinical features as our case [12]. However, our case differed from theirs in the absence of constitutional symptoms of tuberculosis.

Overall, we presented a rare skeletal tuberculosis case in an adult. In this case, it is stressed that such rare clinical presentations should be reported in the literature, which will not only help the treating physicians but will also help modify the management.

## Conclusions

A rare case of a 22-year-old Indian unmarried female who came with complaints of a swollen right index finger is presented. In the absence of a history of tuberculosis and trauma, a very high index of suspicion was required to diagnose and initiate management in this immunocompetent case. The mainstay of treatment was anti-tubercular drugs for a 12-month duration.
